# A dataset of Sattriya dance: Classical dance of Assam

**DOI:** 10.1016/j.dib.2023.109878

**Published:** 2023-12-09

**Authors:** Chayanika Sarmah, Parismita Sarma

**Affiliations:** Department of Information Technology, Gauhati University, Guwahati, Assam 781014, India

**Keywords:** Double handed mudra, Segmentation, Machine learning, Deep learning, ResNet-50, Watershed algorithm, CNN

## Abstract

Sattriya is an Indian classical dance of Assam composed of complex hand gestures, body moments, facial expressions and background music. At a first glance for common people meaning of mudras incorporated in Sattriya dance become difficult to understand. Authors have generated a dataset of Sattriya double handed gestures performed by total fifty artists from different dance academies which can be used to explore ways to build an automated system using machine learning and deep learning algorithms. There are eight double handed gestures or mudras made available through the referred dataset. Mendeley platform is used to store the dataset and samples were collected by visiting some protruding Sattriya dance schools located in Guwahati, Assam. In this article, various tables are used to illustrate variations of the mudras in terms of appearance, title, and compared to other Indian classical dances. To segment and analyze the mudras watershed segmentation is used. In this article researchers have enumerated a few very specific characteristics of Sattriya mudra that may be helpful to academics working in various disciplines of study.

Specifications Table

**Table:** 

Subject	Artificial Intelligence
Specific subject area	The dataset of double handed mudras of Sattriya dance can be especially employed in the development of an automatic recognition system that makes use of deep neural networks and machine learning methods.
Data format	Raw
Type of data	Image
Data collection	Digital samples of Sattriya dance mudras were captured with video of at least 20–30 s duration from the source location using DSLR Canon Camera (EOS 1200 D). Videos were recorded with colored background to reduce injected noise during photo capturing and films. This is an easy and feasible method to segregate the raw videos into frames later. At least three to five videos of each mudra were captured from where the best was selected as video sample.
Data source location	Video samples were collected from some renown Sattriya dance academy situated at Guwahati, Assam, India. All these places were visited by the researchers and captured a number of videos for each of the mudras from different angles. Most significant sources of Sattriya dance school contributed to our work are as follows: • Chandamukha Sattriya Dance Academy, Bamunimaidan, Guwahati-21,Assam,India. • Gandharba Kala Kendra,Hengerabari,Guwahati-781036,Assam,India • Mrs. Parismita Bora, Geetanagar, Guwahati-781021,Assam,India. • Mrs. DreamlyGogoi(Guru of Gandharba Kala Kendra), Gandharba Kala Kendra, Hengerabari,Guwahati-781036,Assam,India. The dataset was stored and analyzed inInstitute: Department of Information Technology, Gauhati University.City: Guwahati, State: Assam, Country: India
Data accessibility	Repository name: Mendeley Data Data identification number: 10.17632/sjvrbxrvh8.2 Direct URL to data: https://data.mendeley.com/datasets/sjvrbxrvh8/2

## 1 Value of the Data

It is possible to develop an automatic system from this dataset that may be used to spot and digitally identify a performer's flaws in real-time Sattriya dance, drama (also known as ‘Bhaona’) and pictures.

In the referred dataset an image set of double handed mudras of Sattriya dance along with their names, three different views of each mudra are uploaded. This will help the researchers to perceive every possible narration of the mudras from three different angles Right, Left and Front. By employing CNN to forecast mudra, this will aid artificial neural networks in their learning process.

The double-handed Sattriya dance sample set aids in comparison research and may assist develop a larger Indian classical dance mudra dataset.

## 2 Data Description

The videos of the hand mudras were captured from front side and the camera was rotating 180 from left to right and again from right to left. Table 1 below shows double handed mudras along with their names made available in Mendeley platform with title “Sattriya-08 Double Handed Mudra's Dataset”.

**Table 1:** Image of double handed mudras along with name, meaning and usage.

Sl no	Name	Image	Meaning	Usage
1	Bardhaman		Crescent	It indicates half-moon.
2	Dol		Swinging arms	Identifies tiredness, boat, hard work.
3	Gajadanta		Tooth of elephant	It recognizes boat and elephant.
4	Kaput		Dove	Lord Shiva, Birth.
5	Karkat		Crab	It represents Crab and Conch shell.
6	Makar1		Fish	Describes monster and animal like elephant, tiger,lion,elephant etc.
7	Makar2		Sea creature	Monster and it also describes seacreature .
8	Samput		Deep thought	Anger

In this work we have used several transformations to increase the sample size such that our dataset becomes voluminous and ready for deep learning model. Brightness adjustment, zooming in and out, shifting and rotations were performed as transformation on the sample set. The augmentation performed on rotation parameter is rotation _range = 7. It means during augmentation an image can be randomly rotated between any degree ranged from +7 to −7. Horizontal and vertical flip was performed on the samples. Vertical flips implies that object's left side becomes right and vice versa. Similarly horizontal flip means flipping along the horizontal axis. Shear_range = 0.1 was applied which performed shearing of the images within a range of +0.1 to −0.1 or −10 % to +10 %. The brightness parameter was augmented as brightness_range=[0.8,1.0], it means that brightness of the samples can be adjusted randomly within the said range. Except for a small number, every mudra displayed in this publication was pre-processed and had back drop removed. Several samples are identified as having body part in the background since they were halted near to the performer's torso and thus making background removal challenging.

The eight double handed Sattriya dance mudras supplied as part of our dataset are displayed in Table 1 above. We have included three views of each mudra front, right and left. Each mudra's name, image, meaning and application are shown in Table 1.

### 2.1 Similarity with other Indian classical dances

A couple of the mudras used in Sattriya dance are unique to it, while others resemble with mudras used in other Indian classical dances (most notably Bharat Natyam). Sattriya dance is profoundly influenced and developed in ‘Sattras’(which are residencies for followers of Sankardev's Ekasarana dharma, the religion he founded) [1,2]. Hence mudras in Sattriya dance depicts characters from Hindu myths basically Lord Bishnu. Most of the mudras used here resembles with mudras of Odissi, Bharat Natyam and Kathakali. Some of them are comparable in appearance but with different identification. On the other hand some are very specific to only Sattriya dance. Table 4 shows three such mudras which are similar in hand gesture but different in title, ie. “Makara2” in Sattriya but “Matsya” in Bharat Natyam. The sources of these samples are available in [3], [4], [5]. A few mudras were artistically created by the renowned Assamese polymaths Madhabdev and Srimanta Sankardev. These unique mudras convey precise meaning within themes of Sattriya dance, contributes to identity and helps in conveying heritage of Assam. Table 2 below shows three such mudras “Bardhaman”, “Gajadanta” and “Makar1”.

**Table 2:** Three double-handed distinctive mudras from Sattriya.

Name	Image
Bardhaman	
Gajadanta	
Makar1	

The two mudras “Dol” and “Samput” in Table 3 below have the same title in Sattriya and Bharatnatyam, although their expression differs on average. Table 4 on the other hand displays three mudras with similar expressions but distinct names; the table itself lists the names of the mudras in Sattriya and Bharat Natyam.

**Table 3:** Identical-sounding but expression-different mudras [6].

Name	Mudra in Sattriya	Mudra in Bharat Natyam
Dol		
Samput

**Table 4:** Mudras with a comparable sentiment but a little title change.

Name in Sattriya	Name in Bharat Natyam	Mudra in Sattriya	Mudra in Bharat Natyam
Kaput	Kaputh		
Karkat	Karkata		
Makar2	Matsya		

## 3 Experimental Design, Materials and Methods

The digital samples of Sattriya dance mudras were captured with video of at least 20–30 s duration from the source location using DSLR Canon Camera (EOS 1200 D). Videos were recorded with coloured background to reduce injected noise during photo capturing and films. This is an easy and feasible method to segregate the raw videos into frames later. At least three to five videos of each mudra were captured from where the best was selected as sample video.

### 3.1 Data acquisition

The sample collection phase was started in the month of July 2022 and ended in November 2022. In this research work plenty of field works were carried out at different Sattriya dance academy situated in Guwahati, Assam, India. The primary sources of sample were “Chandamukha Sattriya Academy”, “Gandharba Kala Kendra”, dance instructor Mrs. Parismita Bora and Mrs. Dreamly Gogoi (Guru of Gandharba Kala Kendra). Utmost care have been taken to film the dancer from her left side to her right side at the moment of recording. Now it was easy to segregate the image frames from the raw video into three most prominent segments called Left, Right and Front. The refereed dataset uploaded in Mendeley platform consists of all these three types of images for each recommended mudra.

After collection the samples were pre-processed. More than one video for each mudra for each dancer were taken from more than one dance academy and merged under the same folder. Thus we ensure variability in the dataset. During pre-processing authors have performed Watershed segmentation technique on the sample set.

### 3.2 Pre-processing

The frames are stored as image frames (.png format) against each mudra after being converted from video clip to image. These images are the final dataset and can be used for classification using machine learning algorithms and Convolution Neural Network. The platform uses OpenCV library functions. We have prepared 08(eight) classes of mudras and collected around 2400 images. Each mudra class contains 300 to 350 samples. All eight mudras are depicted in their original form with backgrounds as shown in Table 5. The watershed algorithm was applied on each of the sample and achieved a bit good result when applied to images without background. Table 5 shows resultant images of all the eight mudras when applied watershed segmentation technique.

**Table 5:** Segmented image using Watershed algorithm (with and without background).

Name	Original Image(with background)	Segmented Image(with background)	Segmented image(without background)
Bardhaman			
Dol			
Gajadanta			
Kaput			
Karkat			
Makar1			
Makar2			
Samput			

### 3.3 Watershed segmentation algorithm

Depending on constraint of the application, availability of resources and image description, watershed segmentation techniques was applied on the source dataset. The watershed technique simulates floods on a picture by placing seed points (markers) in strategic locations to generate basins based on the gradient of the image [7]. It is an effective method for picture segmentation, particularly when the forms of the objects are complicated and erratic. This algorithm did not perform well for all the samples. Although most samples were successfully segmented without backdrop, several flaws are found in mudras like Dol and Makar1.

In order to determine quality of the segmentation results, it is crucial to evaluate the performance of watershed technique. The effectiveness of watershed segmentation may be accessed using varieties of methods and measures. Visual inspection is one of them and we have adopted this technique for performance analysis. The segmented images were visually reviewed and compared to the ground truth and expectations. In this method researchers looked for over segmentation, under segmentation and other artefacts. From Table 5 above it is understandable that watershed segmentation captures all the objects in the frame when background is not removed. This is the reason why Dol and Makar1 are challenging and could not be segmented up to the mark with watershed segmentation. Both of these mudras have to pause very close to the performer's body and background removal becomes quite difficult. Since background removal is unfeasible, segmentation is thus insufficient for them.

We used deep neural networks like ResNet-50 and Convolution Neural Network (CNN) which performed brilliantly with a trustworthy solution. Direct application of the CNN and ResNet 50 networks to the data set produced good accuracy and loss results. The resultant accuracy and loss curves along with the performance tables for both the models are shown below.

Skin color-based segmentation is one of the segmentation methods that may be used on the double-handed mudra dataset. It works well to recognize finger color from background. Background subtraction is another approach which can recognize fingers by subtracting background which are static by nature. It may be useful for video frames. Another segmentation method that incorporates geometrical characteristics including aspect ratio, finger position [8].

## 4 Train the Samples Using Deep Neural Network

### 4.1 Convolution neural network

A deep learning architecture called Convolutional Neural Network (CNN or ConvNet) is primarily made for processing and analyzing visual input, such as pictures and videos. It has performed remarkably well in a variety of computer vision tasks, such as picture segmentation, object identification, and image classification. Each CNN model consists of Input layer, Convolutional layer, Activation function, Pooling layer, Dense layer, Normalization layer, Flatten layer and Output layer [9]. The result obtained by training the double handed Sattriya dance dataset is satisfactory and are shown in Table 6. On the other hand Table 7 shows evaluation of CNN model in terms of accuracy, precision and recall. Figs. 3 and 4 show the accuracy and loss curves of the trained CNN model. The proposed CNN model architecture is shown in Table 10 with respect to hidden layers, flattening layers, filter size etc.

**Table 6:** Evaluation of CNN model performance.

Epoch	Loss	Accuracy	Validation Loss	Validation accuracy
1/40	2.0434	.1840	1.9431	.2587
2/40	1.7183	.3630	1.7993	.3511
3/40	1.2104	.4377	1.5741	.4374
4/40	1.2638	.5180	1.5924	.4271
5/40	1.0399	.6202	1.3179	.5318
6/40	.9354	.6375	1.5580	.4600
7/40	.8592	.6863	1.2716	.5524
8/40	.7654	.7229	1.0885	.5852
9/40	.6589	.7524	0.9362	.6304
10/40	.6207	.7682	1.0677	.6530
11/40	.5178	.8114	.8608	.7187
12/40	.5059	.8058	.9749	.7125
30/40	.1731	.9390	.6895	.8090
31/40	.1959	.9309	.4979	.8563
32/40	.1709	.9451	.5023	.8337
33/40	.1519	.9497	.3374	.8871
34/40	.1638	.9446	.5061	.8439
35/40	.1340	.9568	.5191	.8419
36/40	.1349	.9542	.6083	.8604
37/40	.1127	.9573	.7206	.8645
38/40	.1292	.9563	.5342	.8604
39/40	.1201	.9578	.5789	.8542
40/40	.1212	.9680	.5082	.8932

**Table 7:** Evaluation of CNN model in terms of accuracy, precision and recall.

No. of epoch	Accuracy	Precision	Recall
1	.3017	.3805	.1853
2	.6279	.8895	.4626
3	.8175	.9171	.7629
4	.8836	.9292	.8491
5	.9239	.9279	.9124
6	.9454	.9477	.9368
7	.9425	.9421	.9353
8	.9325	.9382	.9167
9	.9468	.9522	.9440
10	.9454	.9480	.9425
11	.9526	.9553	.9511
12	.9483	.9482	.9468
13	.9339	.9378	.9310
14	.9397	.9421	.9353
15	.9626	.9640	.9626
16	.9382	.9436	.9382
17	.9583	.9610	.9569
18	.9497	.9496	.9483
19	.9440	.9439	.9425
20	.9569	.9583	.9569
21	.9526	.9540	.9526
22	.9497	.9497	.9497
23	.9468	.9468	.9454
24	.9497	.9496	.9468
25	.9540	.9540	.9540
26	.9450	.9553	.9526
27	.9454	.9536	.9440
28	.9454	.9480	.9425
29	.9440	.9452	.9425
30	.9425	.9437	.9397
31	.9540	.9566	.9511
32	.9411	.9449	.9368
33	.9483	.9481	.9405
34	.9511	.9510	.9483
35	.9540	.9553	.9526
36	.9497	.9524	.9483
37	.9555	.9555	.9555
38	.9569	.9583	.9569
39	.9540	.9540	.9526
40	.9626	.9640	.9646

### 4.2 ResNet-50 architecture

**Fig. 1:**
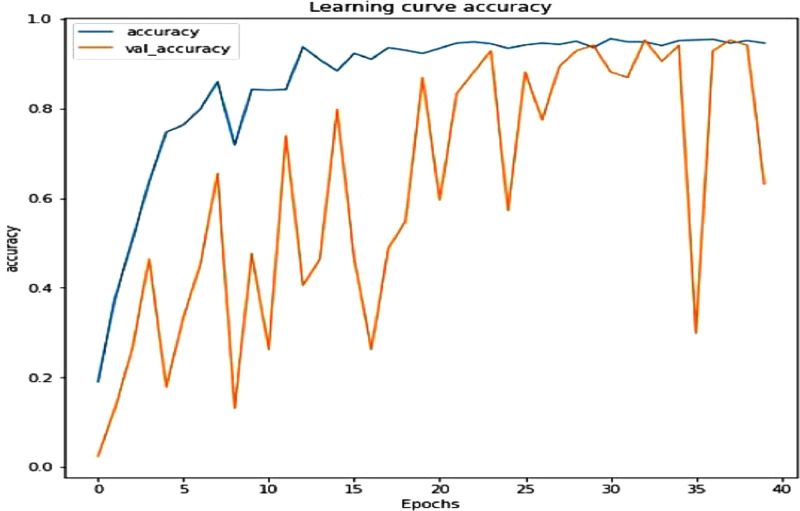
Curves of ResNet −50 model’s accuracy.

**Fig. 2:**
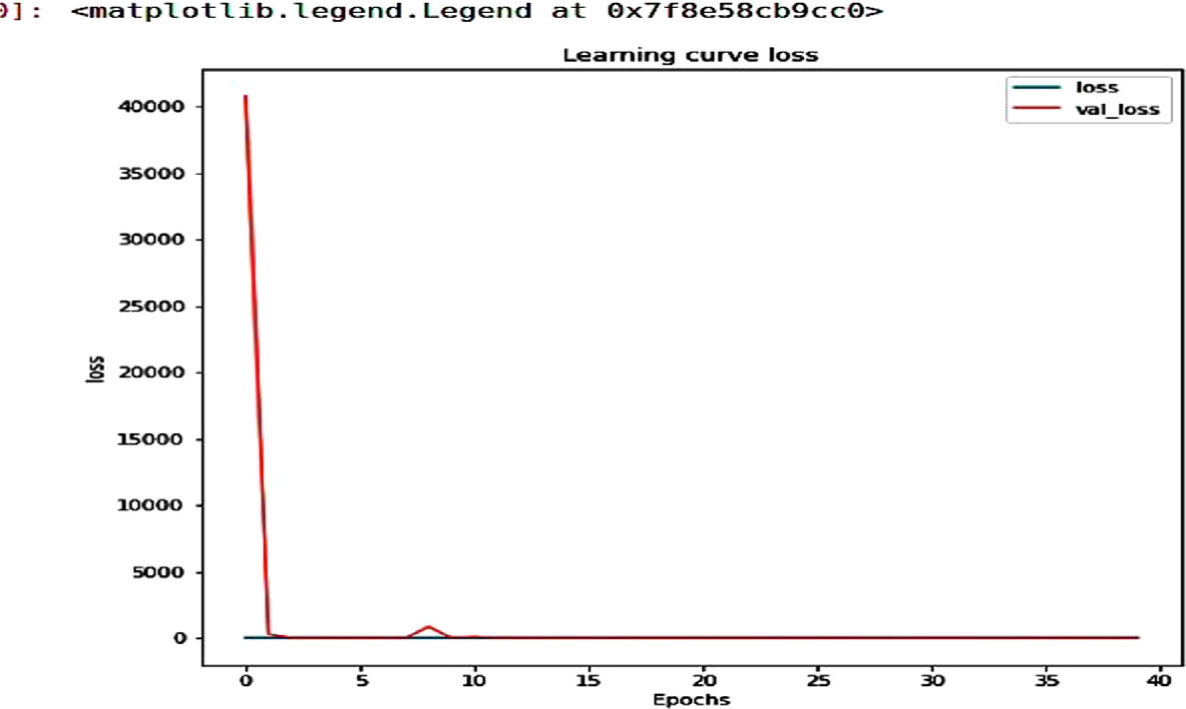
ResNet-50 model loss curves.

ResNet-50 is a convolutional neural network architecture with 50 layers that is a member of the Residual Networks (ResNets) family of deep neural networks [10]. It offers a unique method for expanding a CNN’s convolutional layer structure without encountering the vanishing gradient problem. It is possible due to the skip connections seen in ResNet 50 model that allows the network to bypass certain layers. In this research ResNet 50 model is used to train the mudra dataset and achieved quite good accuracy. As shown in Table 8, we were able to get 94.05 % validation accuracy at 39th epoch. Fig. 1 shows accuracy curve while Fig. 2 shows the loss curve of ResNet-50 model. The performance tables have been reduced in size by the removal of many rows in between. Table 9 shows evaluation of ResNet 50 model in terms of accuracy, precision and recall.

**Table 8:** Evaluation of ResNet-50’s performance.

No of epoch	Loss	Accuracy	Precision	Validation loss	Validation accuracy	Validation precision
1/40	3.7209	0.1897	0.2308	40,767.3008	0.0238	0.0238
2/40	1.5984	0.3764	0.4330	277.1470	0.1310	0.1341
3/40	1.4330	0.5043	0.6366	1.9043	0.2619	0.5238
4/40	1.1375	0.6365	0.7467	1.7796	0.4643	0.5455
5/40	0.8756	0.7471	0.8098	6.9203	0.1786	0.1786
6/40	1.0622	0.7629	0.8010	3.7577	0.3333	0.3415
7/40	0.5734	0.7989	0.8462	3.1493	0.4524	0.4286
8/40	0.5904	0.8592	0.8858	2.2523	0.6548	0.6548
9/40	1.0412	0.7184	0.7682	834.7560	0.1310	0.1310
10/40	0.6418	0.8420	0.8663	5.0156	0.4762	0.4819
11/40	0.6946	0.8405	0.8654	77.9122	0.2619	0.2593
12/40	0.5065	0.8420	0.8584	1.4819	0.7381	0.7531
29/40	0.1004	.9497	0.9511	0.1259	0.9286	0.9398
30/40	0.1321	.9353	0.9380	0.0974	0.9405	0.9405
31/40	0.0965	.9555	0.9582	0.4691	0.8810	0.8916
32/40	0.0983	.9483	0.9494	0.5112	0.8690	0.8690
33/40	0.1108	.9483	0.9481	0.0850	0.9524	0.9524
34/40	0.1062	.9397	0.9410	0.3462	0.9048	0.9036
35/40	0.1104	.9511	0.9509	0.1982	0.9405	0.9405
36/40	0.1196	.9526	0.9538	10.1289	0.2976	0.3049
37/40	0.0945	.9540	0.9540	0.1001	0.9286	0.9398
38/40	0.947	.9400	0.9400	0.0810	0.9504	0.9504
39/40	0.934	.9511	0.9538	0.1120	0.9405	.9405
40/40	0.1008	.9454	0.9493	0.3385	0.6310	.6543

**Fig. 3:**
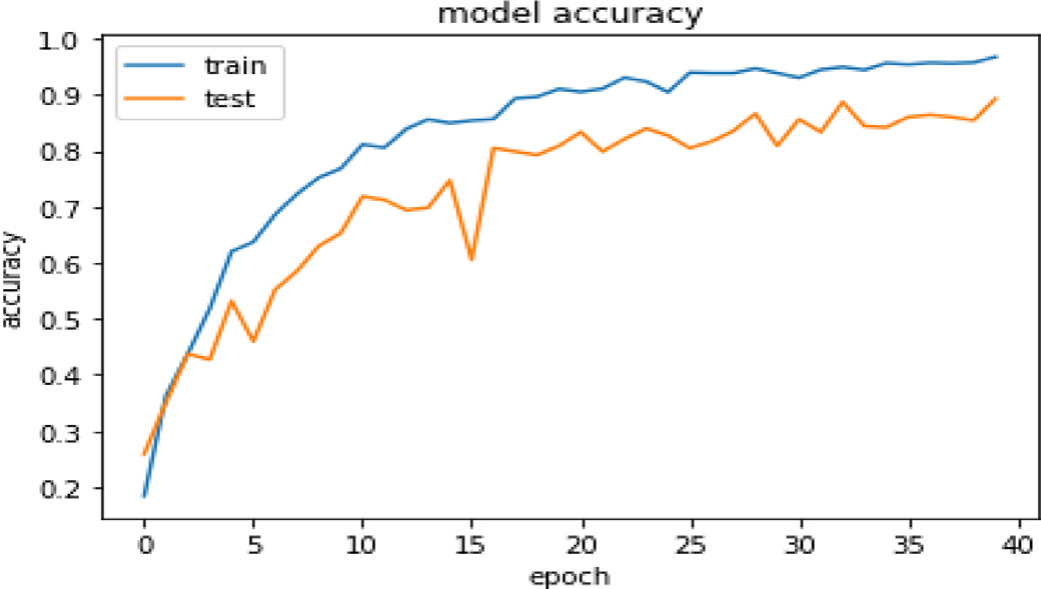
CNN model accuracy curves.

**Fig. 4:**
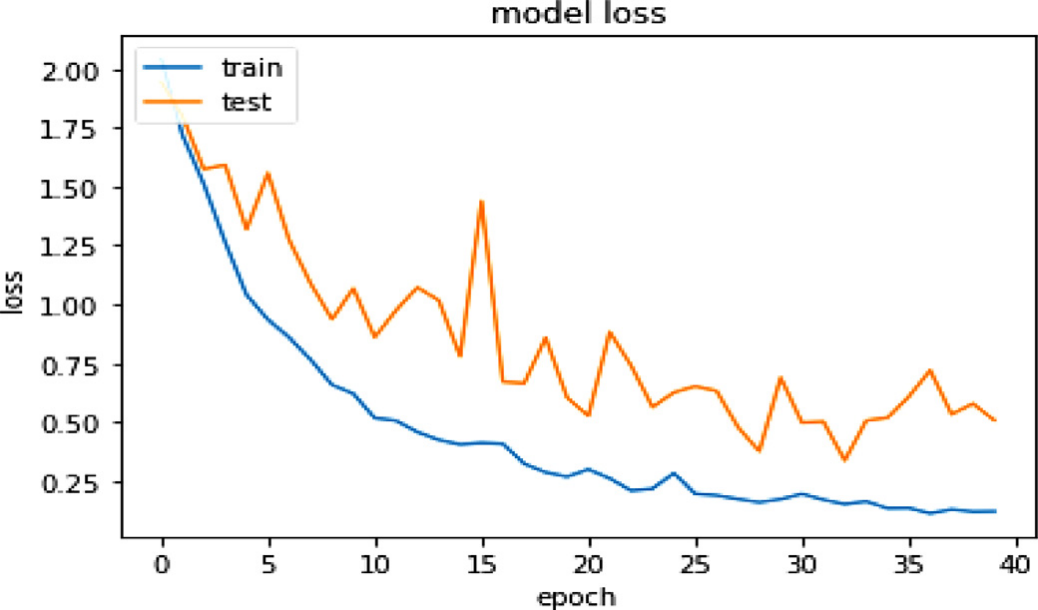
CNN model loss curves.

**Table 9:** Evaluation of ResNet 50 model in terms of accuracy, precision and recall.

No. of epoch	Accuracy	Precision	Recall
1	.2586	.3095	.1121
2	.4353	.68	.1954
3	.4454	.6214	.217
4	.4971	.6836	.3477
5	.6796	.791	.5546
6	.7529	.8388	.7026
7	.7744	.8169	.7241
8	.8060	.8341	.7658
9	.8405	.8725	.8060
10	.8822	.8917	.875
11	.8951	.9044	.8836
12	.9023	.9080	.8927
13	.8908	.8972	.9270
14	.9210	.927	.9153
15	.9095	.9153	.9145
16	.9124	.9145	.9343
17	.921	.9343	.9423
18	.9425	.9423	.9437
19	.9411	.9437	.9423
20	.9411	.9423	.9353
21	.9339	.9353	.9452
22	.9425	.9452	.9336
23	.931	.9336	.9307
24	.9282	.9307	.9336
25	.9310	.9336	.9267
26	.9425	.9448	.9296
27	.9425	.9465	.9353
28	.9497	.9496	.9411
29	.9425	.9439	.9483
30	.9425	.9468	.9425
31	.9483	.9451	.9468
32	.9468	.9425	.9397
33	.9555	.9566	.9425
34	.9454	.9454	.9454
35	.9555	.9582	.9511
36	.9497	.9508	.9454
37	.9569	.9595	.9540
38	.9583	.9583	.9440
39	.9526	.9595	.9526
40	.9569	.9583	.9569

The CNN and ResNet-50 both the networks were trained with total 40-epoch, the CNN-based model has attained the maximum training accuracy 96.8 % and 89.32 % validation accuracy. ResNet-50, on the other hand, shows a maximum 95.11 % training accuracy and 94.05 % validation accuracy at 39 epoch after training with 40 iterations. The performance of both the classification models can be better understood from Table 11 below.

**Table 10:** Summary table of CNN ModelType: sequential.

Layer (type)	Output shape	Activation function	Param #
conv2d_18 (Conv2D)	(None, 62, 62, 32)	ReLU	896
max_pooling2d_18 (MaxPoolin g2D	(None, 31, 31, 32)	ReLU	0
conv2d_19 (Conv2D)	(None, 29, 29, 64)	ReLU	18,496
max_pooling2d_19 (MaxPoolin g2D)	(None, 14, 14, 64)	ReLU	0
conv2d_20 (Conv2D)	(None, 12, 12, 128)	ReLU	73,856
max_pooling2d_20 (MaxPoolin g2D)	(None, 6, 6, 128)	ReLU	0
flatten_8 (Flatten)	(None, 4608)	ReLU	0
dense_16 (Dense	(None, 128)	ReLU	589,952
dropout_2 (Dropout)	(None, 128)	ReLU	0
dense_17 (Dense)	(None, 8)	ReLU	1032

**Table 11:** Summary performance table of both the models.

Technique used (Segmentation+Classification)	Input size (image dimension)	Number of classes	Training accuracy	Val-accuracy
CNN	64, 64, 3	8	96.8 %	89.32 %
ResNet 50	64, 64, 3	8	95.11 %	94.05 %

### 4.3 Data analysis

In this work it is clear that CNN shows better training accuracy but less validation accuracy compared to ResNet-50. In general, more training accuracy but comparatively less validation accuracy occurs due to a number of reasons in machine learning and deep learning algorithms. The most significant problems include overfitting, data mismatch, short datasets, and bias in the data.

Over fitting occurs when a model learns to match training data too closely and broad patterns are replaced by noise and outliers. As a result, it does well with training data but badly with validation data that hasn’t been seen. CNNs are known to be prone to overfitting because of their complexity and huge number of parameters. The likely hood of overfitting increases with network depth and parameter count because the network may acquire generalizable characteristics via memorization of the training set rather than by observation. On the other hand ResNet-50 architecture by the use of residual or skip connections helps in mitigating overfitting in a number of ways like feature reuse, training of deeper networks etc.

In case of data mismatch the datasets used for training and validation may not always be representative of one another. These datasets may differ significantly, in which case the model may perform well on the training data but poorly on the validation data.

If the dataset is relatively small the model may struggle to generalize from the few examples in the training data. In such circumstances, the model may learn the training data too thoroughly and have trouble generalizing to new data.

Another cause is bias in data. if the training data is biased or unbalanced the model may learn to predict the majority class well but perform badly on the minority class. This might result in a lower validation accuracy and a greater training accuracy.

Here while training on CNN model, limited data volume and model overfitting were the main contributors to higher training accuracy and lower validation accuracy. Even though we employed data augmentation prior to training, more data with variation is still required to increase the validation accuracy. On the other hand ResNet-50 shows quite a satisfactory training accuracy as well as improved validation accuracy too at 39 epoch.

### 4.4 Performance comparison with other Indian classical hand mudra dataset

The whole referred dataset was segregated into front, left, and right side perspectives. This was done in order to have a more varied perspective on the samples and to view them from different angles. A comprehensive technique for detecting Sattriya dance mudras might be made using this dataset. Sample variances must be used while training the CNN model in order to produce a reliable, error-free recognizer. Other datasets and learning algorithms with good accuracy are also available for traditional Indian dance mudra. An extensive dataset containing many Indian traditional dance mudra was trained using a few learning methods, according to the article [11]. Researchers of that experiment trained an extremely complicated dataset on a CNN-based model, yet their identification rate was just 93.33 %. Other classifiers like VGG, SVM and Deep ANN were able to get only 87.10, 75 % and 84.7 % accuracy respectively on that same dataset. Another paper [12] describes how CNN and ResNet- 34 based models classify five Indian classical dances included in ICD dataset namely Bharatanatyam, Odissi, Kathak, Kathakali and Yakshagana. They discovered that the recommended model, which could more accurately classify Kathakali dance than other forms, gave the least accuracy to the Bharatanatyam dance style. In this case the CNN model was able to out performs the ResNet-34 model. They employed less samples than required for a deep neural network, that is why this occurred. Accuracy may be raised by increasing the number of training epochs and volume of sample sets. Their model was able to get 78.88 % average accuracy. On the other hand using CNN model we were able to achieve 96.8 % and 89.32 % training and validation accuracy, 95.11 % training and 94.05 % validation accuracy using ResNet-50 model.

## Limitations

This data article basically shows double handed mudras of Sattriya dance which are not easily available on common platform. The researchers of this article collected these mudras of their own, so it is unique and purely original. Our samples are analogous to hand gesture and that is the reason why we selected performers with background knowledge on Sattriya dance. In this case a perfect dancer and a sophisticated digital camera are main possessions while collecting the samples. While capturing photographs from various perspectives and environments, the image's look varies, which might affect the model's performance while recognising the mudras. This is the main limitation on this dataset. Moreover the sample size of double handed mudras used here for segmentation and classification are relatively small, it may affect the performance of a generalized model.

There is scope to improve the dataset in future. Increased number of performers, collection of long time and short time videos, including more classes with noisy samples are some of the possible future improvement of the sample set. The above mentioned extensions of the present data will definitely increase efficiency of classification models.

## Ethics Statement

The sample collection work described here are taken from different dance academy situated at Guwahati, Assam. All the samples were collected with full support and self-willingness of the performers. All the images included in this article were collected by the researchers themselves, no image or species is taken from any electronic media, books, papers, journals etc.

## Citation of Our Mendeley Dataset

CHAYANIKA, CHAYANIKA SARMAH (2023), “Sattriya-08 Double Handed Mudra's Dataset”, Mendeley Data, V2, doi:10.17632/sjvrbxrvh8.2.

## Declaration of generative AI and AI-assisted technologies in the writing process

During the preparation of this work the author(s) used **Paraphrasing Tool - QuillBot AI** in order to improve English and readability.

After using this tool/service, the author(s) reviewed and edited the content as needed and take(s) full responsibility for the content of the publication.

## CRedit Author Statement

**Chayanika Sarmah:** Data Acquisitions, Data Curator, Methodology, Software, **Parismita Sarma:** Data Analysis,Conceptualization, Methodology, Writing, Project Administration.

## Data Availability

Sattriya-08 Double Handed Mudra's Dataset (Original data) (Mendeley Data)
